# Association between face mask use and risk of SARS-CoV-2 infection: Cross-sectional study

**DOI:** 10.1017/S0950268823001826

**Published:** 2023-11-13

**Authors:** Ingeborg Hess Elgersma, Atle Fretheim, Petter Elstrøm, Preben Aavitsland

**Affiliations:** 1 Centre for Epidemic Intervention Research, Norwegian Institute of Public Health, Oslo, Norway; 2 Faculty of Health Sciences, Oslo Metropolitan University, Oslo, Norway; 3 Division of Infection Control, Norwegian Institute of Public Health, Oslo, Norway; 4 Pandemic Centre, Department of Global Public Health and Primary Care, University of Bergen, Bergen, Norway

**Keywords:** coronavirus, epidemics, health policy, infectious disease control, public health

## Abstract

We examined the association between face masks and risk of infection with SARS-CoV-2 using cross-sectional data from 3,209 participants in a randomized trial exploring the effectiveness of glasses in reducing the risk of SARS-CoV-2 infection. Face mask use was based on participants’ response to the end-of-follow-up survey. We found that the incidence of self-reported COVID-19 was 33% (aRR 1.33; 95% CI 1.03–1.72) higher in those wearing face masks often or sometimes, and 40% (aRR 1.40; 95% CI 1.08–1.82) higher in those wearing face masks almost always or always, compared to participants who reported wearing face masks never or almost never. We believe the observed increase in the incidence of infection associated with wearing a face mask is likely due to unobservable and hence nonadjustable differences between those wearing and not wearing a mask. Observational studies reporting on the relationship between face mask use and risk of respiratory infections should be interpreted cautiously, and more randomized trials are needed.

## Introduction

Public health authorities in many countries have recommended, mandated or both, the use of face masks to reduce the spread of COVID-19. This study examines the association between self-reported face mask use and the risk of infection with SARS-CoV-2 in data obtained from a randomized trial on the effectiveness of using glasses in the community against the risk of infection with SARS-CoV-2.

Literature on mask effectiveness in respiratory infection prevention is growing, but their use is still controversial, as demonstrated by the variation in recommendations on face mask use across countries and states [1]. The most recent Cochrane review on the effectiveness of physical interventions in interrupting or reducing the spread of respiratory viruses stated that ‘wearing masks in the community probably makes little or no difference to the outcome of laboratory‐confirmed influenza/SARS‐CoV‐2 compared to not wearing mask’, but the authors also pointed out that ‘the low to moderate certainty of evidence means our confidence in the effect estimate is limited, and that the true effect may be different from the observed estimate of the effect’ [2]. In controlled settings, mechanistic studies suggest that when masks are worn correctly, the risk of infection should be strongly reduced [3]. Studies based on observational data mainly find a negative association between wearing a mask and the risk of COVID-19 infection [4–7]; for example, in their online survey, Xu et al. have found a manifold increase in the risk of infection among the participants who reported not wearing a face mask [8]. In a similar study by Kwon et al., self-reported ‘always’ use of face mask outside the home was associated with around a 65% reduced risk of predicted COVID-19 [9].

The World Health Organization has recently revised their guideline on infection prevention and control in the context of COVID-19, recommending face mask use to reduce SARS-CoV-2 transmission in certain situations, including ‘when in crowded, enclosed, or poorly ventilated spaces’ [10]. The certainty of the underlying evidence was assessed as low to moderate, and the guideline development group concluded that ‘well-conducted, observational studies and/or RCTs exploring the use of masks versus no masks in various settings (for example, indoor, outdoor, ventilation status) would further clarify outstanding questions concerning mask use in community setting’.

Masks may have at least two types of effects on SARS-CoV-2 transmission. Wearing a mask by an infected individual may prevent spread to others (source control). Wearing a mask may also protect the wearers (protective effect) [11].

In this study we revisit the association between use of face masks and the protection against infection from COVID-19. We examine this relationship by using already collected data from a trial conducted in February–April 2022 exploring the effect of wearing glasses on viral transmission [12].

The primary objective was to examine the association between face mask use and the incidence of infection with SARS-CoV-2 (self-reported) adjusted for all observable confounding variables.

Secondary objectives were to carry out analyses of the association between face mask use and (1) the risk of infection with SARS-CoV-2 (notified to health authorities) and (2) the risk of respiratory infection (self-reported).

## Methods

### Study design

In this study we used previously collected data from our trial on the effectiveness of using glasses in the community against the risk of infection with SARS-CoV-2, which took place from 2 February to 24 April 2022, during which participants were continuously recruited [12]. We redistributed the participants from the two trial arms (glasses use or no use) into three groups based on their retrospective report of the level of face mask use during the study period. The analysis was prespecified [13].

The trial data stemmed from the following sources: (1) end-of-follow-up survey, including items on use of face masks, use of glasses, COVID-19 testing and public transportation during the follow-up period; (2) the Norwegian Surveillance System for Communicable Diseases (MSIS), including date of positive COVID-19 PCR test; (3) the Norwegian Immunization Registry (SYSVAK), including date of COVID-19 vaccination; and (4) personal identification number, including date of birth and sex.

During the study period, the recommendation to wear a face mask changed in Norway. After the arrival of omicron variant in November 2021, public health measures were reintroduced to suppress the epidemic, but were then gradually lifted between 13 January and 12 February 2022. This was followed by a huge wave of intensive viral transmission and record levels of hospitalizations for COVID-19 during January–April. Pre 12 February 2022, face mask use was mandated when it was not possible to retain 1 metre distance in shops, shopping malls, restaurants, public transport, taxis and inside public venues. The mandate also applied to employees unless physical barriers were used. To adjust for any bias which may have arisen due to a time-dependable relationship between wearing a mask and the risk of infection, we control for time in the main model as well as in sensitivity analysis.

During the study period, both antigen tests for home use and PCR testing in test stations or in the ordinary health services were widely and freely available to inhabitants in Norway. Only PCR test results were universally registered in the national surveillance system. In the primary analysis, we relied on self-reported positive COVID-19 test, while we looked at reported (notified) COVID-19 test as a secondary outcome.

### Participants

The following eligibility requirements had to be met by all participants in the original trial:

1. at least 18 years of age,
2. did not regularly wear glasses.
3. owned or could borrow glasses that they could use (e.g. sunglasses),
4. had not contracted COVID-19 in the 6 weeks prior to participation,
5. did not have COVID-19 symptoms when providing consent,
6. willing to be randomly assigned to ‘wear’ or ‘not wear glasses’ outside their home when close to others for a 2-week period,
7. provided informed consent.

Participants were followed for 17 days – from when they completed the consent form until they completed the end-of-follow-up survey.

### Exposure

In the end-of-follow-up survey, we asked the participants about their face mask use during the study period. Participants reported on face mask use by selecting one of six responses to the question ‘How often over the last 2 weeks have you used a face mask when you have been close to others outside your home?’ (1) Always; (2) Almost always (at least 75% of the time); (3) Often (50–75% of the time); (4) Sometimes (25–50% of the time); (5) A few times (up to 25% of the time); and (6) Never.

Owing to fewer responses for some of the categories, in our analysis we combined the response categories into: Always/Almost always; Often/Sometimes; and Almost never/Never. This was prespecified in the protocol.

### Outcomes

The primary outcome was a positive COVID-19 test result (self-reported – days 1–17 of the study period).

Secondary outcomes included (1) a reported positive COVID-19 test result (notified; days 1–17 of study period) and (2) an episode of respiratory infection (self-reported symptoms; days 1–17 of study period), defined as having one respiratory symptom (stuffed or runny nose, sore throat, cough, sneezing or heavy breathing) and fever or one respiratory symptom and at least two more symptoms (body ache, muscular pain, fatigue, reduced appetite, stomach pain, headache and/or loss of smell).

### Statistical analysis

We first display the characteristics of participants according to face mask use. We then estimate the cumulative incidence proportion (i.e. the risk) of each of the outcomes in each of the three groups defined by the frequency of mask use. We compute risk ratios (RRs) and adjusted risk ratios (aRRs) using binomial generalized linear models with log link functions [14] or, when these do not converge, robust Poisson regression [15]. Reporting ‘Almost never’/‘Never’ having used face masks is set as the reference level. We adjust for age (continuous + quadratic term), sex, using contact lenses, having used glasses (Always/Almost always; Often/Sometimes; Almost never/Never), use of public transportation and vaccination status (0, 1, 2, 3+ doses) as well as the share of the follow-up time where face mask use was mandatory.

We prespecified two sensitivity analyses: First, we stratify according to whether face mask use was mandatory in at least parts of the total follow-up time. A Chi-squared test of interaction determines whether the effect of exposure was heterogenous. Second, we add the use of fractional polynomials to our model estimating aRRs in order to address time-varying differences in a person’s background risk of infection. We do this by letting *t* be the time in years since the day before the first participant was enrolled in the trial. We consider fractional polynomials of *t* of maximum degree 2, with powers restricted to the set (-2, -1, -0.5, 0, 0.5, 1, 2, 3). We choose among models using a closed testing procedure [16]. All analyses are conducted in R [17].

Data on face mask use were collected in the end-of-follow-up survey; therefore, all participants who did not respond to this survey are excluded from the analysis. We analyse the data using only complete cases as the number of participants who responded to the face mask question and those who did not respond to other survey questions was small (n = 23, 0.7%).

### Bias

Participants in the study were not randomly assigned to wear or not wear face masks, and they were neither provided with nor encouraged to use face masks. During the study period, official guidelines for face mask use changed, with mandatory use in certain situations. This may have affected the participants’ use of face masks, with some choosing to wear them based on their own assessment of risk and effectiveness.

Additionally, there may be other factors that could confound the relationship between face mask use and study outcomes, such as participants in high-risk professions or with risk factors for severe COVID-19. Both groups may be more or less prone to wear face masks while also observing different social distancing practices than the average population. We also cannot rule out reverse causality, in which those testing positive for COVID-19 were more prone to wear masks afterwards in order to protect others. Finally, there could be an association between the inclination to test and the propensity to wear a face mask.

To address these concerns, we control for those variables that are available to us, and that may confound the relationship between face mask use and risk of infection. We also consider several ways to control for differences in background risk over time, as elaborated above. All analyses were prespecified in the protocol and reporting adheres to the STOBE guidelines on items that should be included in reports of observational studies [18]. However, it is important to interpret the results with caution and not infer that our estimates represent the true causal relationship between face mask use and infection risk.

## Results

### Main results

In total 3,231 participants reported on face mask use in the follow-up survey. However, 23 (0.7%) participants were excluded due to missing responses in the adjusted analysis, leaving a total of 3,209 participants with an average age of 46.9 years (SD 15) and the majority being women (2,129, 66.4%). Over 50% of the participants enrolled within the first 2 days (2 and 3 February 2022). Of the participants, 852 (26.6%) reported using a face mask at least 75% of the time outside their home when near others, 861 (26.8%) reported using a face mask between 25% and 75% of the time, and 1,495 (46.6%) reported using a face mask less than 25% of the time (Table 1).

**Table 1.** Characteristics of participants

Characteristics	Use of face masks		
	Almost/almost never (*n* = 1,495)	Sometimes/often (*n* = 861)	Almost always/always (*n* = 852)
Sex			
Female	930 (62.2%)	605 (70.3%)	594 (69.7%)
Male	565 (37.8%)	256 (29.7%)	258 (30.3%)
Age (mean, SD)	47.8 (15.2)	44.7 (14.7)	47.7 (14.9)
Had COVID-19	146 (9.8%)	54 (6.3%)	28 (3.3%)
No. of COVID-19 vaccines received			
0	45 (3.0%)	15 (1.7%)	22 (2.6%)
1	13 (0.9%)	9 (1.0%)	10 (1.2%)
2	263 (17.6%)	173 (20.1%)	154 (18.1%)
3+	1,174 (78.5%)	664 (77.1%)	666 (78.2%)
Wearing glasses			
Almost never/never	841 (56.3%)	407 (47.3%)	318 (37.3%)
Sometimes/often	194 (13.0%)	122 (14.2%)	94 (11.0%)
Almost always/always	460 (30.8%)	332 (38.6%)	440 (51.6%)
Conduct of COVID-19 test			
Yes, home test and at test station	68 (4.5%)	79 (9.2%)	74 (8.7%)
Yes, at test station	10 (0.7%)	6 (0.7%)	7 (0.8%)
Yes, home test	608 (40.7%)	506 (58.8%)	470 (55.2%)
No	809 (54.1%)	270 (31.4%)	301 (35.3%)

The main findings are summarized in Table 2. The crude estimates show a higher incidence of testing positive for COVID-19 in the groups that used face masks more frequently, with 8.6% of participants having never or almost never used masks, 15.0% having sometimes used masks, and 15.1% having almost always or always used masks reporting a positive test result. The risk was 1.74 (1.38 to 2.18) times higher in those who wore face masks often or sometimes, and 1.75 (1.39 to 2.21) times higher in those who wore face masks almost always or always, compared to participants who reported never or almost never wore masks (reference group).

**Table 2.** Main findings: Primary outcome self-reported COVID-19 infection

Exposure group	Infected/total	Risk (%)	RR (95% CI)	aRR (95% CI)
Almost never/never	129/1495	8.6	Reference	Reference
Sometimes/often	129/861	15.0	1.74 (1.38–2.18)	1.33 (1.03–1.72)
Almost always/always	129/852	15.1	1.75 (1.39–2.21)	1.4 (1.08–1.82)

*Note*: In each group, there were 129 individuals infected, purely due to chance.

Adjusting for observable confounders, including vaccination status, resulted in more modest results, with a risk of 1.33 (1.03 to 1.72) times higher in those who wore face masks often or sometimes and 1.40 (1.08 to 1.82) times higher in those who wore face masks almost always or always, compared to participants who reported never or almost never wearing masks (reference group).

For the secondary objectives (Table 3), we found that the proportion of registered COVID-19 cases was higher in the groups using face masks, but aRRs showed no statistically significant difference in risk. Similarly, the risk of self-reported respiratory infection was higher among those wearing face masks, but aRRs were only statistically significant for those wearing face masks sometimes or often (1.19, 95% CI 1.06 to 1.34).

**Table 3.** Secondary outcomes

	Reported (notified) COVID-19				Self-reported respiratory infection			
Exposure group	Infected/total	Risk (%)	RR (95% CI)	aRR (95% CI)	Infected/total	Risk (%)	RR (95% CI)	aRR (95% CI)
Almost never/never	48/1,495	3.2	Ref	Ref	491/1,495	32.8	Ref	Ref
Sometimes/often	40/861	4.7	1.45 (0.96–2.18)	0.94 (0.61–1.48)	371/861	43.1	1.31 (1.18–1.46)	1.19 (1.06–1.34)
Almost always/always	40/852	4.7	1.46 (0.97–2.20)	0.99 (0.63–1.55)	333/852	39.1	1.19 (1.06–1.33)	1.13 (0.99–1.28)

### Sensitivity tests

Using second-degree fractional polynomials, we fitted a model where we let time of inclusion in the study be non-linearly associated with the risk of infection, thereby modelling any differences in background risk linked to the population prevalence of infection when the participant entered the trial. With this approach, the risk of self-reported COVID-19 infection when wearing a face mask was more moderate, 1.03 (95% CI 1.00 to 1.06) times higher in those wearing face masks often or sometimes, and 1.04 (95% CI 1.01 to 1.07) times higher in those wearing face masks almost always/always than in participants having worn face masks never or almost never (Supplementary Table S1). Per peer reviewer’s suggestion, we also conducted a post hoc sensitivity analysis where we used fractional polynomial terms for age instead of quadratic terms for age, with the benefit of fractional polynomials being more flexible in terms of modelling non-linearity. The aRRs were identical to those in the prespecified analysis (Supplementary Table S2).

In our second prespecified analysis, in which the sample was split according to whether face mask was mandatory for at least parts of the follow-up period, there was a higher risk associated with wearing face masks in the period where there was no general recommendation on face mask use in force (Supplementary Figure S1); however, a Chi-squared test of interaction was non-significant (*p* = 0.09).

### Patient and public involvement

No patient or member of the public was involved in conducting this research.

## Discussion

In this cross-sectional study of 3,231 participants, we observed that persons who reported wearing a face mask sometimes/often or almost always/always had a 33% (95% CI 3% to 72%) and 40% (95% CI 8% to 82%) higher incidence of self-reported COVID-19 compared to those who never or almost never wore face masks, adjusting for available relevant confounders. Sensitivity analysis showed that when adjusting for differences in baseline risk over time, the risk of wearing a mask was less pronounced, with only a 4% (95% CI 1% to 7%) increase in the incidence of infection with COVID-19 for those wearing face mask almost always or always compared to those never or almost never wearing face masks. Results from secondary outcomes were largely in the same direction, that is, mask-wearing was associated with an increased relative risk of experiencing respiratory symptoms (1.04, 95% CI 1.01 to 1.07), while we found no clear association between mask-wearing and notified COVID-19 cases.

The results contradict earlier randomized and non-randomized studies of the effectiveness of mask-wearing on the risk of infection [4, 9, 19–24]. Most of these studies reported that wearing a face mask reduced the risk of COVID-19 infection. Some observational studies have reported manifold reduction in infection risk [8, 24], while one community-based randomized trial failed to demonstrate a statistically significant reduction in infection risk [25], and one cluster randomized community trial has found only modest reduction [20].

Our findings may be explained by several factors. A major limitation of our study is the non-randomized, cross-sectional study design. It may be that our participants were more prone to wear masks to protect others from their own infection. This reverse causality may explain the positive association between risk of infection and mask usage and could be supported by the finding that participants reporting wearing masks also were more likely to test themselves for COVID-19. Furthermore, there may be other behavioural differences related to the perception of risk [26] or occupation, which we did not observe, that are linked to the likelihood of wearing mask [27] or being tested for COVID-19 when symptomatic. There is also the possibility that mask wearers feel somewhat protected and thus change their behaviours to not observe social distancing, so that any benefit of masking is offset by increased exposure. Lastly, our main outcome was based on self-report, which is also a possible source of bias.

## Conclusion

We examined the association between face mask use and the incidence of SARS-CoV-2 infection in data obtained from a randomized trial on the effectiveness of using glasses to reduce infection risk. Our findings suggest that wearing a face mask may be associated with an increased risk of infection. However, it is important to note that this association may be due to unobservable and non-adjustable differences between those wearing and not wearing a mask. Therefore, caution is imperative when interpreting the results of this and other observational studies on the relationship between mask wearing and infection risk. Recommendations to wear face masks in the community are largely informed by low certainty evidence from observational studies [10]. More randomized trials or quasi-experimental studies are needed to improve our insights on the effectiveness of face masks for protection against the transmission of respiratory pathogens.

## Supporting information

Elgersma et al. supplementary material Elgersma et al. supplementary material

## Data Availability

The datasets generated and/or analysed in the current study are not publicly available due to them containing personal data, but will be made available by the corresponding author upon reasonable request provided the data is anonymized according to the Norwegian Data Protection Authority guide on anonymization of personal data.

## Supplementary material

For supplementary material accompanying this paper visit http://doi.org/10.1017/S0950268823001826. click here to view supplementary material
