# Supplementary material for: Association between face mask use and risk of SARS-CoV-2 infection: Cross-sectional study
Source: Epidemiol Infect. 2023 Nov 13;151:e194. doi: 10.1017/S0950268823001826 (PMC10728967; doi:10.1017/S0950268823001826)
Supplement: Elgersma et al. supplementary material [file S0950268823001826sup001.docx]

## **Epidemiology and Infection**

## Association between Face mask use and Risk of SARS-CoV-2 Infection – Cross-sectional study.

## Elgersma, A. Fretheim, P. Elstrøm, P. Aavitsland.

Supplementary material

*Supplementary Table S1: Results from sensitivity analysis with fractional polynomials for time*

| Exposure group | Adjusted risk ratio (95% CI) |
| --- | --- |
| Almost never / Never | Reference |
| Sometimes / Often | 1.03 (1.00 - 1.06) |
| Almost always / Always | 1.04 (1.01 - 1.07) |

*Supplementary Figure S1: Analysis of association between wearing a face mask (almost never/never as reference and group) and incidence of self-reported COVID-19 during and after the face mask mandate was in place
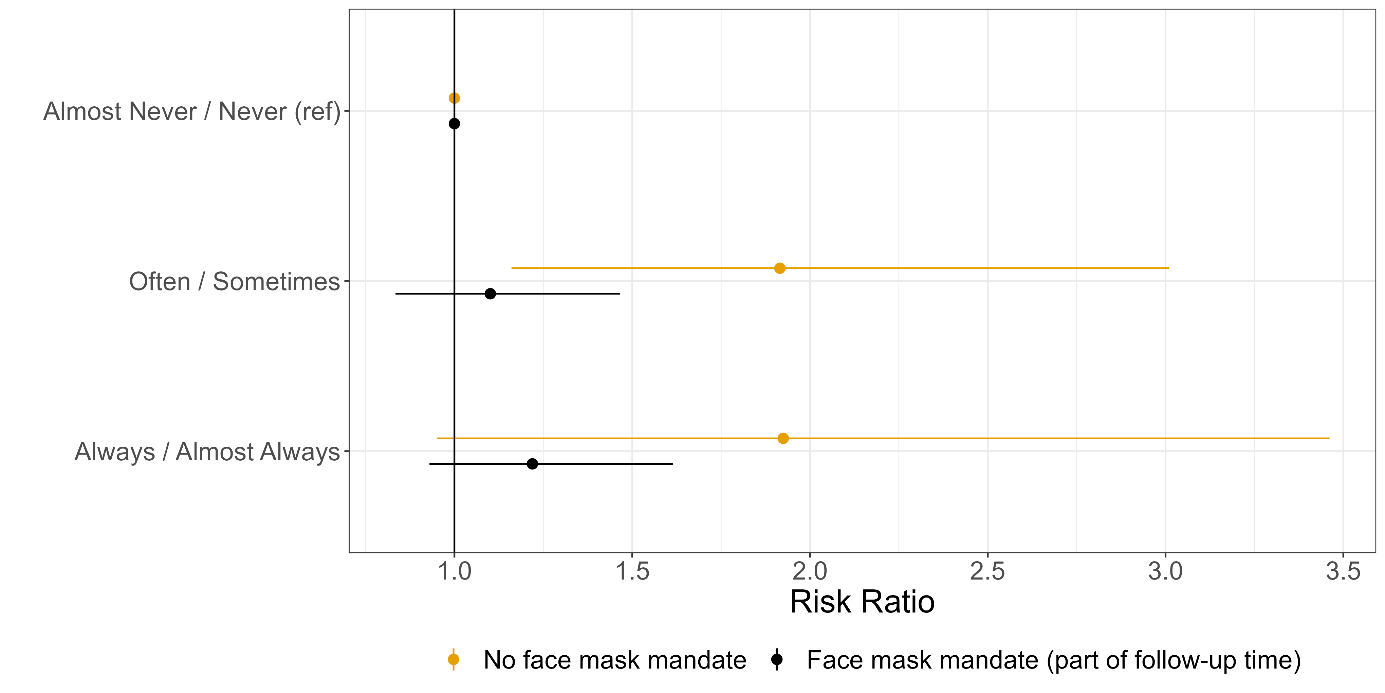
*

| Exposure group | Adjusted risk ratio (95% CI) |
| --- | --- |
| Almost never / Never | Reference |
| Sometimes / Often | 1.03 (1.00 - 1.06) |
| Almost always / Always | 1.04 (1.01 - 1.07) |

*Table S2: Sensitivity analysis suggested by reviewer. Results from sensitivity analysis with fractional polynomials for time and age*

*Note: Peer reviewer suggested to use fractional polynomial terms for age instead of quadratic terms for age, with the benefit of fractional polynomials being more flexible in terms of modelling non-linearity. Table S2 shows the results of this analysis. The aRRs are identical to that in the prespecified analysis(see table S1).*
